# *BDP1* Variants I1264M and V1347M Significantly Associated with Clinical Outcomes of Pediatric Neuroblastoma Patients Imply a New Prognostic Biomarker: A 121-Patient Cancer Genome Study

**DOI:** 10.3390/diagnostics11122364

**Published:** 2021-12-15

**Authors:** Xiaoqing Li, Lan Sun, Andres Stucky, Lingli Tu, Jin Cai, Xuelian Chen, Zhongjun Wu, Xuhong Jiang, Shengwen Calvin Li

**Affiliations:** 1 Department of Oncology, the People’s Hospital of Bishan District, Chongqing 402760, China; 2018020067@stu.cqmu.edu.cn (X.L.); ls_000@usc.edu (L.S.); 2 Department of Hepatobiliary Surgery, the First Affiliated Hospital of Chongqing Medical University, Chongqing 400042, China; 3 Department of Otolaryngology, Keck School of Medicine, University of Southern California, Los Angeles, CA 90033, USA; astucky@usc.edu (A.S.); tulingli425@gmail.com (L.T.); xuelianc@usc.edu (X.C.); 4 Department of Oral and Maxillofacial Surgery, Zhuhai People’s Hospital, Zhuhai Hospital Affiliated with Jinan University, Zhuhai 519000, China; caijin70@163.com; 5 Department of Health Management, Zhuhai People’s Hospital, Zhuhai Hospital Affiliated with Jinan University, Zhuhai 519000, China; 6 Neuro-Oncology and Stem Cell Research Laboratory, Center for Neuroscience Research, CHOC Children’s Research Institute, Children’s Hospital of Orange County (CHOC), 1201 West La Veta Ave, Orange, CA 92868-3874, USA; shengwel@uci.edu; 7 Department of Neurology, Irvine School of Medicine, University of California, 200 S Manchester Ave Ste 206, Orange, CA 92868, USA

**Keywords:** *BDP1*, variant, neuroblastoma, prognostic biomarker, prognosis

## Abstract

Background: Neuroblastoma (N.B.) is the most common tumor in children. The gene *BDP1* (B Double Prime 1) plays a role in cancers but is less known in N.B. Thus, we conducted this study to investigate the value of *BDP1* mutations in N.B. prognosis. Methods: A dataset of 121 NB patients from the Cancer Genome Atlas database was used to analyze *BDP1* gene mutations by RNA sequencing. Kaplan-Meier estimates were performed for overall survival (O.S.) analysis on *BDP1* variants, and Cox’s proportional hazards regression model was used for multivariate analysis. Results: In 121 NB patients, we identified two variants of *BDP1* associated with N.B., located at chr5:71511131 and chr5:71510884. The prevalence of these *BDP1* variants, I1264M and V1347M, was 52.9% (64/121) and 45.5% (55/121), respectively. O.S. analysis showed a significant difference between subgroups with or without *BDP1* variants (*p* < 0.05). Multivariate analysis further revealed that *BDP1*ariants were independent prognostic variables in N.B. (*p* < 0.05). Conclusion: Our results suggest *BDP1* variants are associated with significantly improved clinical outcomes in N.B., thus providing clinicians with a new tool.

## 1. Introduction

As the most common extracranial pediatric solid tumor, ever since defined as an embryonal tumor of the autonomic nervous system, 90% of neuroblastoma (N.B.) cases are diagnosed before the patient is 5-year old, with a median age at diagnosis of around 18 months [1]. Such a delayed diagnosis results in patients with high-risk N.B. who come below 50% for 5-year survival rates [2], especially in older children, with poor outcomes despite aggressive multimodal therapy [2]. This situation sparks the International Neuroblastoma Risk Group (INRG) to standardize diagnostic systems using age, histology, stage, tumor grade, and genomic factors. The genomic factors include DNA ploidy, MYCN (n-myc) status, somatic and germline mutations, as determined by whole-exome, whole-genome [3], Whole-transcriptome sequencing (WTS, also known as RNA-seq) [4], to define high-risk N.B. age and signature-associated driver alterations. The long non-coding RNA MIAT (myocardial infarction associated transcript) regulating neuroblastoma and glioblastoma cell fate [5] acts as an oncogene, potentially predictive, and prognostic biomarker. Some base excision repair pathway variants tighten N.B. susceptibility into a population of eastern Chinese children [6]. Following that, an eight-center case-control study of 898 cases and 1734 controls reveals the single-nucleotide polymorphisms (SNPs) in the METTL14 gene could predispose to N.B. susceptibility [7]. Specifically, the associations with increased N.B. susceptibility for rs9884978 G>A and rs4834698 T>C, the protective effect of rs298982 G>A and rs62328061 A>G, as well as the predisposing effect of rs4834698 T>C are found in specific subgroups. Similar functional polymorphisms manifested at *ERCC1/XPF* genes [8]. Ongoing efforts search for spatiotemporally-expressed biomarkers at the transcriptomic and protein expression level, aimed at diagnosis subtyping of neural-crest-derived malignancy before birth or in early childhood to avoid the metastatic dissemination [9]. These transcriptomic and protein expression patterns might define the genetic, morphological, and clinical heterogeneity. Such a combination of cellular phenotypes and molecular profiles sets up the frameworks of tumor functional context, i.e., tumor microenvironment [10] such tumor tissue elasticity [11].

Along the line, we focus on the human gene *BDP1* (B Double Prime 1), located on chromosome 5q13, encodes a subunit of RNA Polymerase III Transcription Initiation Factor IIIB (*TFIIIB*) [12], which is suppressed by *BRCA1* [13]. However, neither the BDP1’s physiology nor its pathology in humans is fully known, unlike *Drosophila melanogaster* [14,15]. The manuscript attempted to energize the novelty of *BDP1* in N.B., even though the concept of *BDP1*’s cancer-involved is emerging with colorectal cancers [16,17], lung cancer [18], and breast cancer [19]. To date, the importance of BDP1 mutations in N.B. has remained relatively unstudied. Thus, we conducted this study to explore the value of *BDP1* mutations in N.B. by RNA-seq, yielding a new perspective on a pediatric brain tumor N.B.

## 2. Methods

### 2.1. Dataset

A dataset representing 121 NB patients was extracted from The Cancer Genome Atlas (TCGA) database (https://www.genome.gov/Funded-Programs-Projects/Cancer-Genome-Atlas, assessed on 6 October 2020) -The Cancer Genome Atlas (TCGA) was a joint effort of the United States National Cancer Institute (NCI) and the US National Human Genome Research Institute (NHGRI). The Cancer Genome Atlas (TCGA), a landmark cancer genomics program, molecularly characterized over 20,000 primary cancer and matched normal samples spanning 33 cancer types (https://www.cancer.gov/about-nci/organization/ccg/research/structural-genomics/tcga, accessed on 6 October 2020).These patients all completed a long-term clinical observation period from three months to ten years past diagnosis. We downloaded RNA-seq data on these tumor samples to conduct a risk analysis of the death of N.B. patients.

### 2.2. Variant Calling for Detecting BDP1 Variants

We followed the standard variant calling process of GATK ([20] (GATK website www.broadinstitute.org/gatk, accessed on 6 October 2020, for each patient’s RNA-seq data, followed by variant annotation and detection of unique genes/variants in N.B. First, according to the variant calling of GATK, we used Picard to make a duplicate and then used GATK to split reads into exon samples. After that, we ran base recalibration according to the common SNPs and indels. Then, we used the HaplotypeCaller tool in GATK to call variants and filter variants with criteria of D.P. > 10, Q.D. < 2, F.S. > 30, and no SNPcluster. The filters automatically applied by HaplotypeCaller built-in tools of evaluation metrics came with the abbreviations defined as the depth of informative coverage for each sample (D.P.), false-positive (F.P.), FisherStrand (F.S.), QualByDepth (Q.D.), true positive (T.P.). We used AnnoVar [21] for BDP1 variant annotations and only analyzed exonic variants. To study the *BDP1* gene, we generated gene-sample and variant-sample matrices for the 121 samples. Each row represented a sample in the gene-sample matrix. Each column represented a gene from the set (the set of genes of filtered exonic variants). If *BDP1* had exonic variants in a sample, the corresponding cell was assigned the value 1 (or otherwise 0). Each row represented a sample for the variant-sample matrix, and each column represented a gene variant. Similar to the gene-sample matrix, if a *BDP1* variant occurred in a sample, the corresponding cell was assigned the value 1. Otherwise, it was assigned 0.

### 2.3. Statistical Analysis

Overall survival (O.S.) was defined as the time from diagnosis to disease-related death or the date of the last follow-up. For O.S. calculation, patients alive at the end of the study period died from other causes or lost to follow-up were excluded in the Kaplan-Meier analyses and compared using the log-rank test. Cox’s proportional hazards regression model was used for multivariate analysis.

## 3. Results

### 3.1. BDP1 Variants Found in 121 NB Patients

To tie in the clinical relevance, we investigated 121 NB patients to search for the prevalence rate of *BDP1* variants, I1264M and V1347M, showing 52.9% (64/121) and 45.5% (55/121), respectively. Notably, the rate of non-*BDP1* variants was 44.6% (54/121), while the percentage of patients harboring both was 43.0% (52/121). We followed up with characteristics for correlation of these variants with the clinical outcomes.

### 3.2. Clinical Outcomes of N.B. Patients Carrying BDP1 Variants

**Figure 1:**
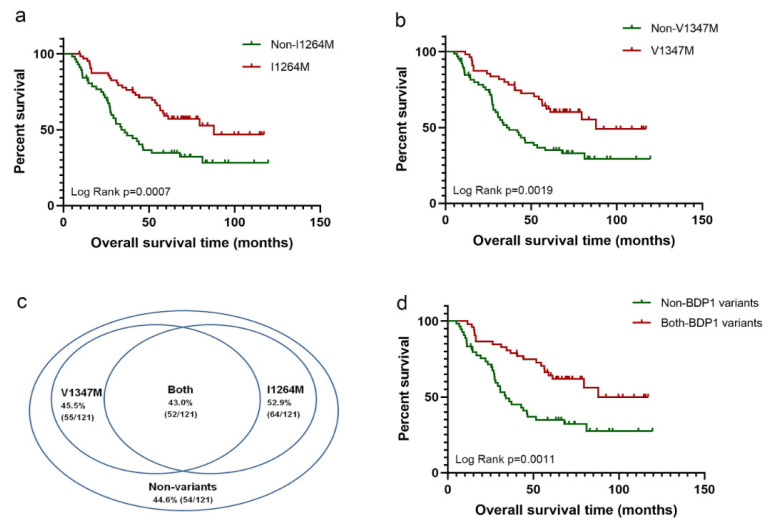
Association of two variants of *BDP1*, I1264M, and V1347M, with clinical outcomes in 121 pediatric neuroblastoma patients. Kaplan-Meier overall survival (O.S.) curves for 121 NB patients according to their *BDP1* variant status. Red and green indicate variant and non-variant, respectively. Two variants of *BDP1*, I1264M (**a**) and V1347M (**b**), significantly improved the overall survival time. Notably, the percentage of patients without *BDP1* variants was 44.6%, while 43.0% of patients harbored both (**c**). There was a significant difference in O.S. between them as well (**d**).

We compared the clinical outcome in subgroups with specific variant patterns, biological markers, and clinical characteristics, including MYCN-amplification, age, gender, stage, Children’s Oncology Group (COG) risk score, and grade in this large, independent population (Table 1). Specifically, we found two variants in BDP1, I1264M and V1347M, significantly associated with improved clinical outcomes (Figure 1). The median O.S. of the patients without I1264M was 35.8 months, and those without V1347M, considerably shorter than the median O.S. of those patients with variant I1264M (87.9 months, *p* = 0.0007) or patients with V1347M (87.9 months, *p* = 0.0019).

Table 1 suggests that I1264M alone has a dynamic prognostic value. Moreover, the median O.S. of the subset with non-*BDP1* variants was 33.8 months, much shorter than the median O.S. of the subset harboring both BDP1 variants (87.9 months, *p* = 0.0011).

### 3.3. BDP1 Variants May Serve for Neuroblastoma Prognosis

We conducted a multivariate Cox hazard analysis of risk factors for N.B. prognosis (Table 2). As expected, stage, ploidy, and age at diagnosis were independent variables significantly associated with O.S. time; *BDP1* variants V1347M and I1264M reduced patients’ risk of death. Noteworthy, *BDP1* variants V1347M and I1264M reduced patients’ risk of death, and the hazard ratios (H.R.) for these variants were 0.488 (95% confidence interval (CI): 0.289–0.826, *p* = 0.007) and 0.58 (95% CI: 0.349–0.964, *p* = 0.035), respectively.

## 4. Discussion

We provide valuable clinical data on a pediatric brain N.B. Specifically, we found that BDP1 variants, I1264M, V1347M, are associated with clinical outcomes, as measured by Kaplan-Meier overall survival (O.S.) curves, extrapolated from 121 patients of the TCGA data. The high-level amplification of *MYCN* on chromosome 2p24 occurs in approximately 20% of N.B. patients, indicating its link to aggressive disease with a poor prognosis. Inhibitors that downregulate *MYCN/MYC* proteins can suppress N.B. tumor growth [22]. Mutations in the tyrosine kinase domain of Anaplastic lymphoma kinase (*ALK*) have also been identified in N.B. FDA approved a series of *ALK* tyrosine kinase inhibitors (TKIs) for use in *ALK*-driven cancers [23]. Furthermore, the paired-like homeobox 2B gene (*PHOX2B*) was discovered as the first N.B. predisposition gene, accounting for 6.4% of heritable cases [24]. Despite the development of molecular strategies, the diagnostic and prognostic value of many gene mutations in N.B. patients is not very clear. Here, we first report the association of *BDP1* mutations with N.B. prognosis, showing that two *BDP1* variants, I1264M and V1347M, are located at chr5:71510884 and chr5:71511131, are significantly correlated with better outcomes in an independent N.B. population. We further verified the prevalence of these *BDP1* variants, I1264M and V1347M, were 52.9% (64/121) and 45.5% (55/121), respectively. Interestingly, the rate of patients harboring both was 43.0% (52/121), suggesting a high rate of overlap between them. Consistently, the minor allele frequency (MAF) of *BDP1* polymorphism variants in an NCBI database were detected by DNA-seq ranging from 17% to 22%, which was similar to our results using RNA-seq.

The weakness of the current approach was to make a comparison by evaluating the survival on the ground of gene expression on a single gene, which might be uninformative, given the fact that the variables that affect the clinical course of these patients are many and deserve an evaluation on a case history with clinical data collected (retrospective or prospective). However, the lack of such case history with clinical data collected (retrospective or prospective) is evident in the N.B.-databases. The solution to this problem might fall on conducting the work on registries in the future, which may be useful on gene expression studies but not on the outcome of the study only. Artificial intelligence-based medicine such as machine learning [25] might integrate case history, clinical information, molecular pathology, whole-genome sequencing, RNA seq., lifestyle courses, treatment-driven pathophysiological, and biomarker changes into comprehensive lifetime management. In previous reports, mutations in genes such as *ALK* or *ATRX* are associated with poor prognosis in N.B. [5]. In contrast, others, such as *TAM1* variants, were associated with improved clinical outcomes [22]. Gene polymorphisms also confer N.B. susceptibility resulting in different clinical outcomes such as *METTL14* [7] and *ERCC1/XPF* [8]. In our study, age at diagnosis (≥18 months), diploidy, and stage IV were all associated with poor prognosis, as expected. Interestingly, both *BDP1* variants significantly improved overall survival time and reduced patients’ risk of death from N.B. Therefore, our findings support that the protective phenotype of *BDP1* variants may exist in subsets of N.B. patients with the following implications.

**Figure 2:**
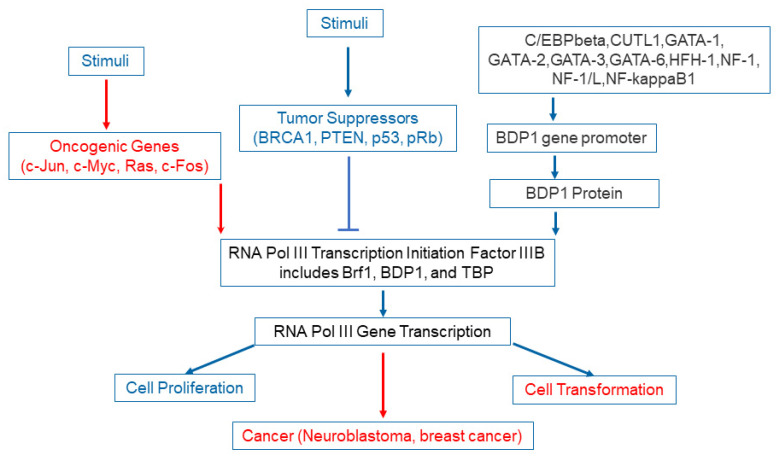
Role of *BDP1* in the TFIIIB complex of transcription machinery in cell proliferation, cell transformation, and cancer progression. TFIIIB is a complex of the transcription machinery of *RNA Pol III* gene transcription [12]. TFIIIB consists of *Brf1*, *BDP1*, *and TBP*. Oncogenic proteins, such as *c-Jun*, *c-Myc*, *Ras*, *and c-Fos* activate *TFIIIB* to enhance *RNA Pol III* gene transcription, resulting in cancer tumorigenesis, while tumor suppressors, such as *BRCA1* [13], *PTEN*, *p53*, and *pRb*, inactivate its activity to decrease the transcription of Pol III genes [14], leading to repression of tumor development [*BDP1*, a.k.a., TATA Box Binding Protein (*TBP*)-Associated Factor] [16]. *TFIIIB*). The concept of *BDP1*’s cancer-involved is emerging with colorectal cancers [16,17], lung cancer [18], and breast cancer [19].

First, establishing the association of BDP1 variants with clinical outcomes warrants in vitro or in vivo experimental verifications. Second, new bioinformatic methodologies may be used to explore the networks of these variants with accuracy. Third, both variants may affect BPD1 mRNA expression levels in subsets of N.B. patients [26]. Fourth, NB heterogeneity surfaces as single-cell characterization of malignant phenotypes and developmental trajectories of adrenal N.B. [27] for subclonal evolution [28]. Lastly, we must expand to explore the mechanisms by which these two variants affect the functions of NB-involved *BDP1* (Figure 2). Thus, the value of *BDP1* mutations in N.B. deserves further investigation.

## Figures and Tables

**Table 1:** Clinical characteristics and variant patterns in NB population (n = 121).

	Patients n = 121 (%)	Overall Survival	
Characteristics		Median OS (months)	*p* Value
**Gender** Male Female	71 (58.7) 50 (41.3)	56.4 58.1	0.816
**Race** White Black Others	87 (71.9) 19 (15.7) 15 (12.4)	58.1 56.4 56.5	0.538
**Location** Abdomen Non-abdomen	106 (87.6) 15 (12.4)	56.5 61.2	0.604
**Grade** Poorly differentiated Not poorly Unknown	94 (77.7) 7 (5.8) 20 (16.5)	79.5 40.4 37.1	0.573
**Ploidy** Diploid Hyperdiploid	50 (41.3) 71 (58.7)	36.7 81.1	0.008 *
**MYCN status** amplified non-amplified	26 (21.5) 95 (78.5)	33.8 58.8	0.248
**Age at diagnosis** <18 months ≥18 months	20 (16.5) 101 (83.5)	NA 44.0	<0.001 *
**Stage** 4 Not 4	100 (82.6) 21 (17.4)	46.1 NA	<0.001 *
**COG Risk** High risk Not high risk	103 (85.1) 18 (14.9)	46.1 NA	<0.001 *
**BDP1** Non- I1264M I1264M	57 (47.1) 64 (52.9)	35.8 87.9	<0.001 *
**BDP1** Non- V1347M V1347M	66 (54.5) 55 (45.5)	35.8 87.9	0.002 *

* Variables were statistically significant by Kaplan–Meier estimates using the log-rank test. NA: Not applicable.

**Table 2:** Multivariate Cox hazard analysis of risk factors for prognosis.

Characteristics	HR	95% CI	*p* Value
**Ploidy** Hyperdiploid Diploid	0.526 1.0 (Ref)	0.317–0.872	0.013
**MYCN status** amplified non-amplified	0.986 1.0 (Ref)	0.531–1.829	0.963
**Age at diagnosis** ≥18 months <18 months	20.692 1.0 (Ref)	1.13–380.07	0.041
**Stage** Not 4 4	0.148 1.0 (Ref)	0.03–0.729	0.019
**COG Risk** High risk Not high risk	6.824 1.0 (Ref)	0.319–145.76	0.219
**BDP1** I1264M Non- I1264M	0.580 1.0 (Ref)	0.349–0.964	0.035
**BDP1** V1347M Non-V1347M	0.488 1.0 (Ref)	0.289–0.826	0.007

Abbreviations: HR, Hazard Ratio; CI, confidence interval.

## Data Availability

Publicly available datasets were analyzed in this study. This data can be found here: https://portal.gdc.cancer.gov.
